# Utilization of the emergency department by kidney transplant recipients: a retrospective cohort study from a high-volume transplant center

**DOI:** 10.1097/MS9.0000000000000481

**Published:** 2023-04-11

**Authors:** Belal Nedal Sabbah, Mohammad Alghafees, Ahmad Nedal Sabbah, Tarek Ziad Arabi, Saleha Abdul Rab, Abdulaziz Mohammed Alaklabi, Hytham Mubarak Abdalla, Ahmed Essam Maklad, Mazin Ibrahim El Sarrag, Emad Sameer Hawari, Omar Hussien Barbour, Ahmed Khedr, Faisal Alrasheed, Mohammed Alshalhoub, Abdulrahman Altamimi, Ghali Sayedahmed, Khalid Alshuwaier, Yasser Alkharashi, Abdulrahman Albassam, Salman Bin Ofisan

**Affiliations:** a College of Medicine, Alfaisal University; b College of Medicine, King Saud bin Abdulaziz University for Health Sciences; c Emergency Medicine; d Department of Transplantation, King Abdulaziz Medical City, Riyadh; e College of Medicine, Prince Sattam Bin Abdulaziz University. Al-Kharj, Saudi Arabia

**Keywords:** emergency department utilization, emergency medicine, post-transplant care, renal transplantation, saudi arabia

## Abstract

This study aims to assess the trends of emergency department (ED) visits among kidney transplant recipients in a high-volume transplant centre.

**Methods:**

This retrospective cohort study targeted patients who underwent renal transplantation at a high-volume transplant centre from 2016 to 2020. The main outcomes of the study were ED visits within 30 days, 31–90 days, 91–180 days, and 181–365 days of transplantation.

**Results:**

This study included 348 patients. The median (interquartile range) age of patients was 45.0 years (30.8, 58.2). Over half of the patients were male (57.2%). There was a total of 743 ED visits during the first year after discharge. 19% (*n*=66) were considered high-frequency users. High-volume ED users tended to be admitted more frequently as compared to those with low frequencies of ED visits (65.2% vs. 31.2%, respectively, *P*<0.001).

**Conclusion:**

As evident by the large number of ED visits, suitable coordination of management through the ED remains a pivotal component of post-transplant care. Strategies addressing prevention of complications of surgical procedures or medical care and infection control are aspects with potential for enhancement.

Highlights

- Kidney transplant most often visit the emergency department (ED) 6–12 months after discharge.
- Among kidney transplant recipients, high-volume ED users are admitted significantly more than low-volume users.
- High-volume visits are significantly associated with several conditions, such as pneumonia, cardiac dysrhythmias, and urinary tract infections.

Since the first successful renal transplant from a healthy live donor to their identical twin over half a century ago, transplant surgery has emerged as a well-established treatment for end-stage renal disease^1^,^2^. The introduction of effective immunosuppressants has led to a significant increased life expectancy of organ transplant recipients^3^. Due to the significant increase of the transplant recipient population, there is an increased amount of emergency physicians who are faced with caring for this complex population^4^. These complex patients may present with underlying medical conditions that may be associated with their transplant surgeries or immunosuppressive therapies.

In the literature, there are several comprehensive studies evaluating hospital re-admissions and ED visits among renal transplant recipients^5^–^7^. However, there is limited data on the usage of the ED post-renal transplantation in Saudi Arabia and the Middle East. Thus, this study aims to assess the trends of ED visits among kidney transplant recipients in a high-volume transplant centre.

## Materials and methods

This retrospective cohort study targeted all adult patients, aged 14 years and older, who underwent renal transplantation at our hospital from 1 January 2016, to 31 March 2020. Patients who had a second renal transplant, or a renal transplantation in a different hospital and followed-up at our hospital, were excluded.

The data were collected using the BESTCare system (ezCareTech, South Korea). This study included all patients complying with the inclusion criteria in the study period. The main outcomes of this study were ED visits within 30 days, 31–90 days, 91–180 days, and 181–365 days of transplantation. Demographic data, including age, sex, BMI, and co-morbidities were collected. The BMI of each patient was categorized into four groups: <18.5 kg/m^2^ as underweight, 18.5–24.9 kg/m^2^ as normal, 25.0–29.9 kg/m^2^ as overweight, ≥30 kg/m^2^ as obese based on the WHO general population classification^8^. The transplantation-related data collected were donor condition, cold ischaemia time, delayed graft function, postoperative haemodialysis, postoperative ICU admission, and length of stay. ED visit parameters consisted of visit intervals, overall number of ED visits, whether the patient is a high-volume user (four or more annual visits) or not, presenting complaints, and ED admission decisions^9^.

Data analysis was performed using RStudio (R version 4.1.1). We used frequencies and percentages to express categorical variables and median and interquartile ranges to present numerical variables. The statistical differences between high-volume and low-volume users of ED were explored using a Pearson’s χ^2^ test or a Fisher’s exact test for categorical variables or a Wilcoxon rank sum test for numerical variables. Statistical significance was considered at *P*<0.05. This study is registered on the research registry with a unique identifying number: researchregistry8691^10^. This study has been reported in line with the STROCSS criteria^11^. The Institutional Review Board of King Abdullah International Medical Research Center, Ministry of National Guard - Health Affairs, Riyadh, Kingdom of Saudi Arabia, approved the study, with approval number RC20/204/R.

## Results

### Demographic and end-stage renal disease (ESRD)-related characteristics of patients

Three hundred forty-eight patients were included in this study. The median (interquartile range) age of patients was 45.0 years (30.8, 58.2). Over half of the patients were male (57.2%). Almost one-third of patients were overweight (32.6%) and obese (32.6%). The most common causes of ESRD were diabetes mellitus (35.1%), hypertension (30.2%), and glomerular nephritic syndrome (14.3%). The majority of patients underwent haemodialysis at the time of ESRD (82.3%). Living donors represented 77.8% of patients with 0 human leukocyte antigen mismatch number (80.6%). More details about patients’ characteristics are listed in Table 1.

**Table 1:** Demographic and ESRD-related characteristics of patients.

Parameter	Category	*N* (%)
Sex	Male	199 (57.2)
	Female	149 (42.8)
Age	Years	45.0 (30.8, 58.2)
BMI	Underweight	15 (4.3)
	Normal weight	106 (30.5)
	Overweight	113 (32.6)
	Obese	113 (32.6)
	Missing	1
Cause of ESRD	Diabetes mellitus	93 (35.1)
	Polycystic kidney disease	16 (6.0)
	Hypertension	80 (30.2)
	Glomerular nephrotic syndrome	24 (9.1)
	Glomerular nephritic syndrome	38 (14.3)
	Obstructive uropathy	7 (2.6)
	Post-renal acute kidney injury	1 (0.4)
	Pre-renal acute kidney injury	4 (1.5)
	Intra-renal acute kidney injury	2 (0.8)
	Missing	83
Type of dialysis at time of ESRD	Haemodialysis	279 (82.3)
	Peritoneal dialysis	38 (11.2)
	Preemptive transplantation	22 (6.5)
	Missing	9
Donor type	Living	270 (77.8)
	Deceased	77 (22.2)
	Missing	1
HLA mismatch number	0	220 (80.6)
	1	32 (11.7)
	2	21 (7.7)
	Missing	75
Cold ischaemia time	<12 h	338 (97.4)
	12–18 h	6 (1.7)
	18–24 h	2 (0.6)
	>24 h	1 (0.3)
	*Missing*	*1*
Post-transplant ICU admission	Yes	70 (20.4)
	Missing	5
Post-transplant dialysis	Yes	21 (6.1)
	Missing	4
Length of stay after transplant	Days	7.0 (6.0, 11.0)
	Missing	2
Delayed graft function	Yes	24 (6.9)

ESRD, end-stage renal disease; HLA, human leukocyte antigen.

There was a total of 743 ED visits during the first year after discharge. During the year, 20.1% (*n*=70) of patients visited the ED once, 14.1% (*n*=49) visited the ED twice and 8.3% (*n*=29) visited ED three times. Mean number of ED visits was 3.4±3.2. During the first 30 days after discharge, 37.9% (*n*=132) of renal transplant (RT) patients visited the ED. Moreover, 23.6% (*n*=82) utilized the ED in the interval of 31–90 days after discharge, 23% (*n*=80) in the interval of 91–180 days after discharge, and 36.8% (*n*=128) in the interval of 181–365 days after discharge. Of all RT patients that visited the ED in the first year, 19% (*n*=66) were considered high-frequency users. Of all ED visits, 29.8% (*n*=222) led to re-admissions. 52.7% percent of RT patients that visited the ED within the first 30 days after discharge were readmitted. 56.1% that visited within the interval of 31–90 days were readmitted. 65% that visited within the interval of 91–181 days were readmitted. 46.9% that visited within the interval of 181–365 days were readmitted.

### Factors associated with being a high-volume user

High-volume usage of the ED did not differ significantly based patient’s demographic and ESRD-related data. However, high-volume ED users tended to be admitted more significantly compared to those with low frequencies of ED visits (65.2% vs. 31.2%, respectively, *P*<0.001), as shown in Table 2. Notably, comorbid conditions were not significantly different between high-volume and low-volume users (Table 3). Nevertheless, the proportions of presenting complaints were significantly higher among high-volume users compared to their peers; these included abdominal pain (68.2% vs. 35.8%, *P*<0.001), infectious symptoms (60.6% vs. 19.1%, *P*<0.001), urinary symptoms (36.4% vs. 11.3%, *P*<0.001), laboratory abnormalities (39.4% vs. 8.5%, *P*<0.001), neurologic symptoms (28.8% vs. 8.5%, *P*<0.001), cardiovascular symptoms (24.2% vs. 1.8%, *P*<0.001), and other symptoms (48.5% vs. 28.7%, *P*=0.002). Furthermore, high-volume users were more likely to be diagnosed with selected conditions compared to their counterparts, including urinary tract infections (37.9% vs. 12.8%, *P*<0.001), viral infections (19.7% vs. 9.9%, *P*=0.027), abdominal pain (40.9% vs. 11.0%, *P*<0.001), electrolyte imbalance (27.3% vs. 6.0%, *P*<0.001), procedural or care-related complications (13.6% vs. 2.1%, *P*<0.001), fluid-balance disorders (16.7% vs. 3.5%, *P*<0.001), pneumonia (9.1% vs. 1.8%, *P*=0.008), fever of unknown origin (10.6% vs. 1.1%, *P*<0.001), cardiac dysrhythmias (6.1% vs. 0.01%, *P*=0.001), and other diagnostic conditions (62.1% vs. 39.4%, *P*<0.001), as shown in Table 3.

**Table 2:** The association between demographic and ESRD-related data and the intensity of ED visits.

		A high-volume user		
Parameter	Category	No, *N* = 282, *N* (%)	Yes, *N* = 66, *N* (%)	*p*
Sex	Male	163 (57.8)	36 (54.5)	0.630
	Female	119 (42.2)	30 (45.5)	
Age	Years	44.0 (30.0, 58.0)	48.5 (31.2, 60.5)	0.319
BMI	Underweight	10 (3.6)	5 (7.6)	0.165
	Normal weight	92 (32.7)	14 (21.2)	
	Overweight	90 (32.0)	23 (34.8)	
	Obese	89 (31.7)	24 (36.4)	
	Missing	1	0	
Cause of ESRD	Diabetes mellitus	71 (33.5)	22 (41.5)	0.585
	Polycystic kidney disease	15 (7.1)	1 (1.9)	
	Hypertension	62 (29.2)	18 (34.0)	
	Glomerular nephrotic syndrome	19 (9.0)	5 (9.4)	
	Glomerular nephritic syndrome	33 (15.6)	5 (9.4)	
	Obstructive uropathy	6 (2.8)	1 (1.9)	
	Post-renal acute kidney injury	1 (0.5)	0 (0.0)	
	Pre-renal acute kidney injury	4 (1.9)	0 (0.0)	
	Intra-renal acute kidney injury	1 (0.5)	1 (1.9)	
	Missing	70	13	
Type of dialysis at time of ESRD	Haemodialysis	222 (81.3)	57 (86.4)	0.708
	Peritoneal dialysis	32 (11.7)	6 (9.1)	
	Preemptive transplantation	19 (7.0)	3 (4.5)	
	Missing	9	0	
Donor type	Living	221 (78.6)	49 (74.2)	0.438
	Deceased	60 (21.4)	17 (25.8)	
	Missing	1	0	
HLA mismatch number	0	183 (80.6)	37 (80.4)	0.953
	1	26 (11.5)	6 (13.0)	
	2	18 (7.9)	3 (6.5)	
	Missing	55	20	
Cold ischaemia time	<12 h	274 (97.5)	64 (97.0)	0.516
	12–18 h	5 (1.8)	1 (1.5)	
	18–24 h	1 (0.4)	1 (1.5)	
	>24 h	1 (0.4)	0 (0.0)	
	Missing	1	0	
Post-transplant ICU admission	Yes	56 (20.1)	14 (21.5)	0.802
	Missing	4	1	
Post-transplant dialysis	Yes	16 (5.7)	5 (7.8)	0.562
	Missing	2	2	
Length of stay after transplant	Days	7.0 (6.0, 11.0)	8.0 (6.0, 12.0)	0.054
	Missing	1	1	
Delayed graft function	Yes	19 (6.7)	5 (7.6)	0.789
ED visits ending with admission	Yes	75 (31.2)	43 (65.2)	**<0.001**
	Missing	42	0	

ED, emergency department; ESRD, end-stage renal disease; HLA, human leukocyte antigen; HLA, human leukocyte antigen.

Bolded values indicate statistical significance.

**Table 3:** The association between the intensity of ED use and co-morbidities, presenting complaints and final diagnostic conditions.

			A high-volume user		
Parameter	Category	Overall, *N* = 348, *N* (%)	No, *N* = 282, *N* (%)	Yes, *N* = 66, *N* (%)	*p*
Co-morbidities	Hypertension	283 (81.3)	226 (80.1)	57 (86.4)	0.243
	Diabetes mellitus	127 (36.5)	99 (35.1)	28 (42.4)	0.266
	Dyslipidemia	79 (22.7)	60 (21.3)	19 (28.8)	0.190
	Coronary artery disease	36 (10.3)	27 (9.6)	9 (13.6)	0.329
	Smoking	23 (6.6)	19 (6.7)	4 (6.1)	>0.999
	Congestive heart failure	9 (2.6)	6 (2.1)	3 (4.5)	0.380
	Anaemia	40 (11.5)	32 (11.3)	8 (12.1)	0.859
	Arrhythmias	9 (2.6)	6 (2.1)	3 (4.5)	0.380
	COPD	1 (0.3)	1 (0.4)	0	>0.999
	Other	66 (19.0)	51 (18.1)	15 (22.7)	0.386
Presenting complaints	Abdominal pain/ GI symptoms	146 (42.0)	101 (35.8)	45 (68.2)	**<0.001**
	Infectious symptoms	94 (27.0)	54 (19.1)	40 (60.6)	**<0.001**
	Neurologic symptoms	43 (12.4)	24 (8.5)	19 (28.8)	**<0.001**
	Lab abnormalities	50 (14.4)	24 (8.5)	26 (39.4)	**<0.001**
	Urinary symptoms	56 (16.1)	32 (11.3)	24 (36.4)	**<0.001**
	Cardiovascular symptoms	21 (6.0)	5 (1.8)	16 (24.2)	**<0.001**
	Other	113 (32.5)	81 (28.7)	32 (48.5)	**0.002**
Final diagnoses	Complications of surgical procedures or medical care	15 (4.3)	6 (2.1)	9 (13.6)	**<0.001**
	Viral infection	41 (11.8)	28 (9.9)	13 (19.7)	**0.027**
	Lower respiratory disease	70 (20.1)	51 (18.1)	19 (28.8)	0.051
	Infection and inflammation of device; implant; or graft	10 (2.9)	6 (2.1)	4 (6.1)	0.100
	Hypertension with complications	3 (0.9)	1 (0.4)	2 (3.0)	0.093
	Electrolyte imbalances	35 (10.1)	17 (6.0)	18 (27.3)	**<0.001**
	GI conditions	58 (16.7)	31 (11.0)	27 (40.9)	**<0.001**
	Urinary tract infection	61 (17.5)	36 (12.8)	25 (37.9)	**<0.001**
	Fluid-balance disorder	21 (6.0)	10 (3.5)	11 (16.7)	**<0.001**
	Pneumonia	11 (3.2)	5 (1.8)	6 (9.1)	**0.008**
	Septicaemia	3 (0.9)	1 (0.4)	2 (3.0)	0.093
	Fever of unknown origin	10 (2.9)	3 (1.1)	7 (10.6)	**<0.001**
	Hypotension	1 (0.3)	1 (0.4)	0	>0.999
	Cardiac dysrhythmias	4 (1.1)	0	4 (6.1)	**0.001**
	Complication of device	5 (1.4)	3 (1.1)	2 (3.0)	0.241
	Other	152 (43.7)	111 (39.4)	41 (62.1)	**<0.001**

COPD, chronic obstructive pulmonary disease; ED, emergency department; GI, gastrointestinal.

Bolded values indicate statistical significance.

As shown in Table 4, the compliance rate for outpatient nephrology visits ranged between 94% at first 30 days to 95.4% at 31–90 days. Furthermore, Table 5 shows the use of immunosuppression drugs among the patients in our sample.

**Table 4:** Descriptive statistics of outpatient nephrology visits.

Parameter	Category, (days)	*N* (%)
Outpatient nephrology visits	First 30	327 (94.0)
	31–90	332 (95.4)
	91–180	329 (94.5)
	181–365	331 (95.1)

**Table 5:** Post-transplantation immunosuppression drug use by patients.

	Drug	No. patients	% of patients
Post-transplantation immunosuppression	Prednisone	104	31.1
	Tacrolimus	326	97.6
	Mycophenolate mofetil	323	96.7
	Imuran	1	0.3

## Discussion

This study was conducted at our centre over a period of 3 years and 3 months, and included a total of 348 renal transplant recipients. It aims to describe ED visits by these patients focusing on rates, complaints, co-morbidities, re-admissions and ability of the ED to handle such cases.

While the length of stay after transplant was roughly the same for all patients, and delayed graft function was seen only in a small population (6.9%), the number and causes of visits were variable and numerous. Male predominance was seen, with 57.2% males and 42.8% females. This is similar to the American multicenter study, where male constituted around 63.1%^12^. Cumulatively, this may suggest gender related complaints and complications. Though the long-term survival rate for kidney transplant recipients is more in females as compared to males in the Taiwan study, no significance difference was found in our study between males and females in regard to the frequency of ED use^13^. In addition, no significant association of obesity could be seen in the ED visits, as the ratio of normal BMI, overweight, and obese patients was almost the same. Moreover, no association was found with BMI category, which is in accordance with other studies^12^–^14^.

In our population, all the patients who underwent renal transplants at our centre visited the ED at least once during the first-year post-renal transplant. Interestingly, these findings differ from a study that was done in Granada, where less than 50% of the population utilized the ED services during the first-year post-transplantation^15^. This stresses the importance of more scheduled outpatient visits for renal transplant patients, better analgesia, clear discharge instructions, and further evaluation of their concerns^16^.

37.9% of our patients presented within the first month of discharge. This data are similar to a study conducted in the United States in 2014^17^. They reported that 30.5% of their patients visited the ER within a month of after discharge^17^. However, there is a study conducted in Cleveland which has differing data from our study^18^. They reported only 12% of their patients visiting the ED within a month of transplantation^18^. Furthermore, they reported that the most common cause of ED visits was complications of therapeutic procedures (implants, grafts) whereas we reported abdominal pain as the most common cause^18^. These interesting findings shows a variation in the trend of ED utilizations, which may be due to a potential increased incidence of early post-transplant complications in our population, further studies on this manner are warranted.

The rates of ED visits were 23.6% in 31–90 days, 23% in 91–180 days and 36.8% in 181–365 days. An increase in the number of visits by kidney transplant recipients is seen from 6 to 12 months after discharge. This percentage is similar to the Cleveland study, showing a 40% utilization during the same period^18^. As described in their paper, the increased incidence may be because patients prefer the ED as a suitable care unit over an extended period. Thus, there is a need of a proper and sufficient ED setup. In our study there was a total of 29.8% patients that were readmitted owing to their conditions and complications. This is contrary to the Cleveland research, where half the patients had to be readmitted^18^.

Gastrointestinal symptoms and infectious symptoms were the main causes of ED visits, which is consistent with other studies^15^,^19^,^20^. These remain the major reasons for visits due to regular use of immunosuppressive drugs after transplant, which may increase the incidence of urinary tract infections and gastrointestinal symptoms^21^. This data indicates the need to have protocols for the ED staff for dealing with post-kidney transplants patients and their most common presentations.

Co-morbidities such as diabetes and heart failure are an important risk factor of post-transplant complications, graft rejection and mortality^16^,^22^. Nevertheless, there was no association in our population between having co-morbidities and being a frequent ED user. In addition, the high influx of ED visits may be due to patients’ concerns regarding their health post-transplant, this is further shown by a study done by Tucker *et al*.^23^ who reported that 74% of their patients visiting the ED were concerned about quality of life, co-morbidities, and kidney related issues.

This study has several strengths and limitations. One of the strengths is that this study collected data using a reliable and efficient electronic health record system, which allowed for the easy retrieval of data for analysis. Another strength is this study’s focus on a specific and understudied population of renal transplant recipients in Saudi Arabia and the Middle East, which provides valuable information on the trends of ED visits in this region. However, this study also has limitations. One of the limitations is that this study only includes data from a single high-volume transplant centre, which may limit the generalizability of the findings to other transplant centres. Additionally, this study is limited by its retrospective design, which makes it difficult to establish causality between variables.

As another limitation of our study, we acknowledge the lack of data from our specific region to serve as a direct comparison to our findings. While we compared our results to studies conducted in the United States of America, differences in healthcare infrastructure and practices may have influenced the presentation of post-op renal transplant patients in our region. In addition, we acknowledge that we did not report the compliance rate of outpatient nephrology visits for high-volume users and low-volume users separately. This is an important factor that could potentially affect the rate of ED utilization and should have been recorded. We hope that our study serves as a stimulus for further research in our region to address these limitations and provide more comprehensive data on this important topic. Finally, the study is limited by the small sample size, which may limit the ability to detect significant differences between groups.

## Conclusion

The regular visits to the ED and utilization of its facilities warrants establishment of a protocol for post-renal transplantation patients. ED staff should be aware of and trained to deal with these cases, and the department itself should be up to date. Care protocols should be formed and followed, and specific interventions need to be introduced. There is also a need for coordination between the ED and nephrology units. The number of renal transplant recipients is increasing with better transplant facilities. The authors aim to introduce awareness regarding this issue, leading to proper patient care and better healthcare system for patients and medical staff alike, along with mitigating the preventable factors that could increases the financial burden on the healthcare system.

## Ethics approval

IRB approval was waived for this study.

## Consent to participate

Not applicable.

## Source of funding

This study did not receive funding from any source.

## Authors contributions

All authors contributed to the research and/or preparation of the manuscript. B.N.S., M.A. participated in the study design and wrote the first draft of the manuscript. B.N.S., A.N.S., T.Z.A., S.A.R., A.MA., H.M.A., A.E.M., M.I.S., E.S.H., O.H.B., A.K., K.A., Y.A., A.A., and S.B.O. collected and processed the samples. B.N.S., M.A., A.A., and G.S. participated in the study design and performed the statistical analyses. All authors read and approved the final manuscript.

## Conflicts of interest disclosure

The authors declare no conflict of interest

## Research registration unique identifying number (UIN)

NA

## Availability of data and material

Not applicable.

## Code availability

Not applicable.

## Provenance and peer review

Not commissioned, externally peer-reviewed.
